# Virtual screening, optimization, and identification of a novel specific PTP-MEG2 Inhibitor with potential therapy for T2DM

**DOI:** 10.18632/oncotarget.10341

**Published:** 2016-06-30

**Authors:** Meiyan Wang, Xiaobo Li, Lei Dong, Xiubo Chen, Weiren Xu, Runling Wang

**Affiliations:** ^1^ Tianjin Key Laboratory on Technologies Enabling Development of Clinical Therapeutics and Diagnostics (Theranostics), School of Pharmacy, Tianjin Medical University, Tianjin, China; ^2^ Department of Pediatrics, Emory University School of Medicine, Atlanta, GA, USA; ^3^ Tianjin Medical University Eye Hospital, Tianjin, China; ^4^ Tianjin Key Laboratory of Molecular Design and Drug Discovery, Tianjin Institute of Pharmaceutical Research, Tianjin, China

**Keywords:** PTP-MEG2 inhibitor, core hopping, diabetes, selectivity, molecular dynamics simulation, Gerotarget

## Abstract

Megakaryocyte protein tyrosine phosphatase 2 (PTP-MEG2) is a tyrosine phosphatase expressed in megakaryocytic cells, and causes insulin sensitization when down regulated. Therefore, specific inhibitors of PTP-MEG2 are potential candidates for novel Type 2 Diabetes (T2DM)therapy. In this study, we discovered PTP-MEG2 inhibitors using high throughput and virtual screening (HTS/VS) and structural optimization *in silicon*. Eight compound-candidates were identified from the interactions with PTP-MEG2, protein tyrosine phosphatase 1B (PTP1B) and T cell protein tyrosine phosphatase (TCPTP). Results from enzymatic assays show compounds *4a* and *4b* inhibited PTP-MEG2 activity with an IC50 of 3.2 μM and 4.3 μM, respectively. Further, they showed a 7.5 and 5.5 fold change against PTP1B and TCPTP, respectively. We propose compounds *4a* and *4b* are PTP-MEG2 inhibitors with potential therapeutic use in T2DM treatment.

## INTRODUCTION

Protein tyrosine phosphatases (PTPs) are enzymes that remove phosphate groups from substrates, and contain a highly conserved active site motif C(X)_5_R. PTPs play important roles in signaling transduction of intra- and inter-cellular functions associated with cell growth, proliferation, differentiation, survival, apoptosis, adhesion, and motility [1–4]. Megakaryocyte protein tyrosine phosphatase 2 (PTP-MEG2), encoded by protein tyrosine phosphatase non-receptor type 9 (PTPN9) [5, 6], was originally cloned from human endothelial cells and megakaryocytes. Reduction of PTP-MEG2 in hepatic cells increases insulin sensitivity [7]. Therefore, PTP-MEG2 inhibitors with high selectivity and activity represent potentially efficient therapies for Type 2 Diabetes (T2DM).

Classical strategies for inhibitor discovery were designed to identify compounds that target the catalytic site of enzymes. However, acquiring specific PTP-MEG2 inhibitors has been problematic given active sites typically have high sequence homology in the PTP super family Table S1 [8, 9]. According to multiple sequence alignments and conformational superposition of PTP-MEG2 with other PTPs, previous research described an extended binding gorge from the active site as a selective binding pocket that helps identify PTP-MEG2 inhibitors [4].

In this study, we used scaffold-based strategies to discover novel PTP-MEG2 inhibitors *in silicon,* and applied enzymatic assays *in vitro* to evaluate inhibitory activities. ZINC01433265 was screened from the drug-like molecular database through high throughput and virtual screening (HTS/VS) as a lead-compound for further optimization. Eight structure-candidates were identified as potential PTP-MEG2 inhibitors via “core-hopping”. Compounds 4a and 4b were chosen to synthesize for enzymatic assay based on the binding affinity with various PTPs and their chemical structure. Our results show 4a and 4b inhibited the activity of PTP-MEG2 with IC50 of 3.2 μM and 4.3 μM, respectively.

## RESULTS

### Virtual screening and core-hopping

**Figure 1:**
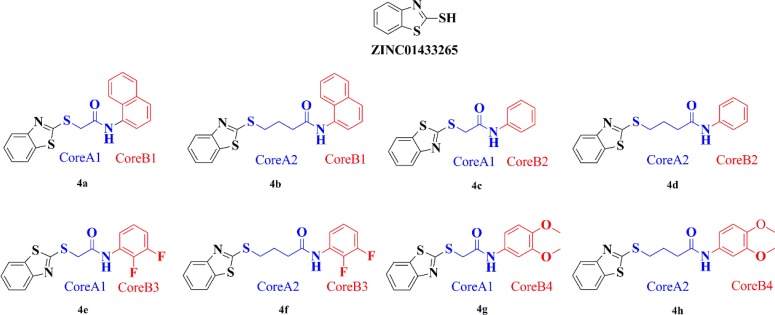
ZINC01433265 derivatives with high docking scores generated by core hopping

Structure-based virtual screening in ZINC drug-like database was performed *in silicon*. According to virtual screening results, molecule ZINC01433265 (Figure 1) was selected as the PTP-MEG2 inhibitor given it fit neatly into the docking pocket of PTP-MEG2 and formed hydrogen bonding with Arg311. However, we realized ZINC01433265 was too small to occupy the entire space of that pocket. Therefore, the small molecule 4a needed to be extended to reach the other side of the catalytic pocket by core-hopping of Schrodinger *in silicon*. The structures of the top 8 ZINC01433265 derivatives obtained from core-hopping are listed in Figure 1 and their docking scores are ranked in Table 1.

**Table 1:** The docking scores and predicted ADME properties of 8 ZINC01433265 derivatives

Rank	No.	Docking scores(Kcal/mol)			Predicted ADME properties			
		PTP-MEG2	PTP1B	TCPTP	PSA^a^	logPo/w^b^	logS^c^	PMDCK^d^
1	**4a**	−9.67	−1.91	−3.26	106	5.4	−6.0	730.4
2	**4b**	−9.58	−1.34	−2.89	183	5.8	−6.2	1282.7
3	**4c**	−9.56	−1.60	−2.11	194	6.0	−6.6	1232.8
4	**4d**	−9.44	−1.10	−1.34	136	5.4	−6.1	713.6
5	**4e**	−9.34	−1.75	−1.24	167	5.3	−5.8	908.6
6	**4f**	−9.14	−1.51	−1.10	144	5.4	−6.0	502.4
7	**4g**	−9.13	−1.53	−1.06	135	5.2	−6.9	1254.3
8	**4h**	−9.13	−1.66	−1.06	165	5.7	−5.3	1232.5

a The van der Waals surface area of polar nitrogen and oxygen atoms (7.0 to 200.0).

b The predicted octanol/water partition coefficient (−2.0 to 6.5).

c The predicted aqueous solubility, where S (mol dm^−3^) is the concentration of the solute in a saturated solution that is in equilibrium with the crystalline solid (−6.5 to 0.5).

d The apparent MDCK permeability (nm/s); MDCK cells are considered as a good mimic for the blood brain barrier. QikProp predictions are for non-active transport (<25 poor; >500 great).

### ADME prediction

The results of ADME prediction obtained from Qikprop of the 8 candidates are shown in Table 1. PSA, logPo/w, logS, and PMDCK were within the acceptable ranges for further drug discovery.

### Synthesis of 4a and 4b

**Figure 2:**
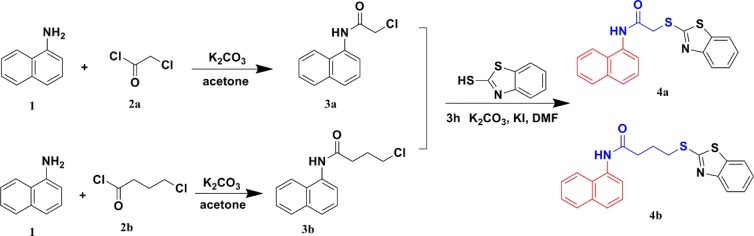
Synthetic routes of compounds 4a and 4b

The synthetic routes of compounds 4a and 4b are shown in Figure 2. Naphthalen-1-amine reacted with chloroacetyl chloride treated with K_2_CO_3_ as the catalyst in acetone to produce 3a. Compound 3a was mixed with benzo[d]thiazole-2-thiol in DMF with K_2_CO_3_ and KI catalyst for 3 hours to generate compound 4a. Compound 4b was synthesized using the same method above:

2-(benzo[d]thiazol-2-ylthio)-N-(naphthalen-1-yl)acetamide(4a) A white solid with the following characteristics: 71.7% yield; ^1^H-NMR (CDCl3, 400 MHz): *δ* 10.05 (br s, 1H), 8.14 (d, *J*=8.0 Hz, 1H), 8.00 (d, *J*=8.0 Hz, 1H), 7.95 (d, *J*=8.0 Hz, 1H), 7.87-7.80 (m, 2H), 7.65 (d, *J*=8.0 Hz, 1H), 7.52-7.30 (m, 5H), 4.26 (s, 2H).

4-(benzo[d]thiazol-2-ylthio)-N-(naphthalen-1-yl)butanamide(4b) A white solid with the following characteristics: 16.9% yield; ^1^H-NMR (400 MHz, CDCl3): *δ* 8.21 (br s, 1H), 7.88-7.86 (m, 3H), 7.74-7.72 (m, 2H), 7.95 (d, *J*=8.0 Hz, 1H), 7.54-7.38 (m, 3H), 7.36-7.21 (m, 2H), 3.58 (t, *J*=6.8 Hz, 2H), 2.75 (t, *J*=6.8 Hz, 2H), 2.36 (m, 2H).

**Table 2:** PTP-MEG2, PTP1B and TCPTP inhibitory activities of compounds 4a and 4b

No.	IC_50_(μM)		
	PTP-MEG2	PTP1B	TCPTP
4a	3.2	>25	>25
4b	4.3	>25	>25

### Enzymatic assays

We found compound 4a inhibited PTP-MEG2 with an IC50 of 3.2 μM, approximately 7.5 fold higher compared with PTP1B and TCPTP. Compound 4b inhibited PTP-MEG2 with an IC50 of 4.3 μM, 5.5 fold higher compared with PTP1B and TCPTP.

### Drug-protein interaction

**Figure 3:**
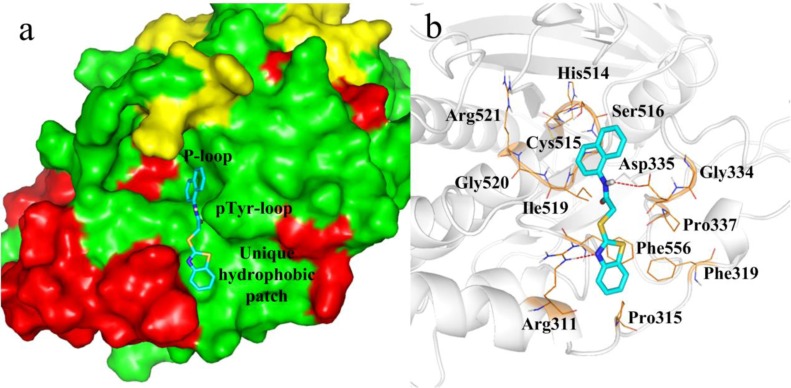
Docking of compound 4a with PTP-MEG2 **a.** Surface representation of PTP-MEG2 in complex with 4a. **b.** Docking interactions of compound 4a with PTP-MEG2 active site. The H-bond interactions between PTP-MEG2 and compound 4a are shown as red dashed lines.

The PTP-MEG2 crystal structure shows the ligand binding domain is surrounded by P-loop (residues514−521), a pTyr recognition loop (residues331−338) and a hydrophobic patch formed by residues Pro315, Phe319, Pro337, and Phe556 (Figure 3). We found the hydrophobic patch was unique and positioned in close proximity to the second aryl phosphate binding site, originally identified in PTP1B. In addition, results show Gly334, located in pTyr loop, was also unique for PTP-MEG2.

### Binding energy

**Figure 4:**
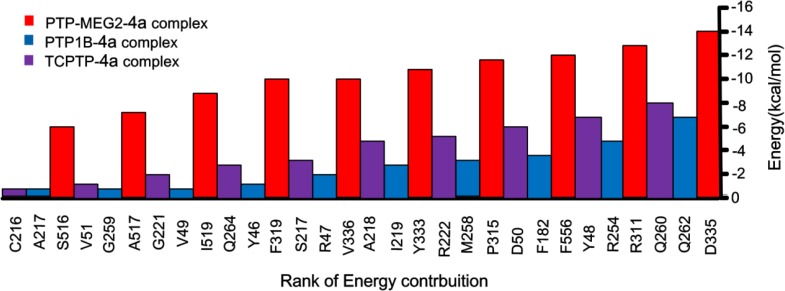
The interaction energies of top 10 pairwise residues of the complex formed by PTP-MEG2 (red), PTP1B (blue), and TCPTP (purple) with compound 4a

Binding energy describes van der Waals interactions and shot range electrostatic interactions between the ligand and receptor. We show the top 10 residues, forming binding energies tightly with 4a within a radius of 11 Å of the ligand, of PTP-MEG2 (red), TCPTP (purple) and PTP1B (blue) in (Figure 4). Results show the drug-protein binding energy in PTP-MEG2-4a system was significantly higher compared with TCPTP-4a and PTP1B-4a, indicating compound 4a has higher affinity with PTP-MEG2. We found Asp335, the chief mediated residue located on pTyr recognition loop, contributed −14.1 kcal/mol to the binding energy in the PTP-MEG2-4a system. In addition, we show other key residues, including Tyr333 (−11.9 kcal/mol) and Val336 (−10.0 kcal/mol), formed steady hydrogen bonding and van der Waals with compound 4a during the simulation. Further, we found other H-bonds between the benzo[d]thiazole ring of compound 4a and Arg311. Finally, at the other side of the binding area, the contribution of the characteristic PTP-MEG2 residues related to PTP-MEG2 specificity were Phe556 (−12.5 kcal/mol), Pro315 (−12.0 kcal/mol) and Phe319 (−10.0 kcal/mol) (Figure 4).

## DISCUSSION

Given that the PTP active site is highly conserved, identifying selective PTP inhibitors that bind to the pTyr-binding cleft is challenging. The PTP active site is highly charged to accommodate the pTyr substrate. Therefore, most existing PTP inhibitors are also highly polar compounds and exhibit poor bioavailability, which do not readily cross cell membranes.

In this study, we discovered PTP-MEG2 inhibitors using HTS/VS and structural optimization *in silicon*. Eight compound-candidates were identified from the interactions with PTP-MEG2, PTP1B, and TCPTP. Results from enzymatic assays show 4a and 4b inhibited PTP-MEG2 activity with an IC50 of 3.2 μM and 4.3 μM, respectively (Figure S1). In addition, we confirmed that PTP inhibitor potency and selectivity was achieved by targeting peripheral residues other than the active site.

We also show the PTP-MEG2 crystal structure of the ligand binding domain is surrounded by P-loop, pTyr recognition loop, and a hydrophobic patch (Figure 3). At the P-loop position, core B1 of compound 4a stretched into the catalytic pocket of PTP-MEG2 (Figure 3a). However, core B1 was too small to perfectly fit the pocket. We plan to further optimized B1 to identify compounds with higher affinity to PTP-MEG2. At the pTyr-loop position, compound 4a formed one H-bond with Asp335 and van der Waals interaction with Tyr333. Although Tyr333 and Asp335 in the pTyr-loop were conserved among the PTPs, this intense interaction may account for the PTP-MEG2 inhibitory activity of compound 4a. In addition, the H-bond formed by the benzo[d] thiazole ring of compound 4a and Arg311 enhanced the binding affinity between PTP-MEG2 and compound 4a (Figure 3b). The hydrophobic binding pocket formed by Pro315, Phe319, Pro337 and Phe556 is unique to PTP-MEG2 compared with other PTPs. The benzo[d] thiazol ring of compound 4a was engaged in strong aromatic stacking interactions with residues Pro315, Phe319 and Phe556 (Figure 3b), which was favorable to the PTP-MEG2 selectivity of 4a.

Compared with the binding energies in PTP-MEG2-4a system, compound 4a had lower binding interactions with TCPTP and PTP1B (Figure 4). This finding suggests compound 4a is a potential candidate for PTP-MEG2 inhibition with high selectivity.

In summary, eight novel ZINC01433265-based PTP-MEG2 inhibitors with high docking scores were designed by the core-hopping method. After synthesis and enzymatic assays, compound 4a exhibited significant PTP-MEG2 selectivity (3.2 μM) over the two high homologous proteins TCPTP (>25 μM) and PTP1B (>25 μM). Molecular dynamics simulation results show 4a bound more steadily to PTP-MEG2 active pocket and formed stronger binding interaction with key residues of PTP-MEG2 compared with TCPTP or PTP1B. Therefore, compound 4a should be considered a potent and specific inhibitor against PTP-MEG2 and a potential therapeutic for T2DM.

## MATERIALS AND METHODS

The crystal structures of PTP-MEG2 (PDB ID: 4GE6) [4, 10] and other PTPs were downloaded from the Protein Data Bank (PDB) of RCSB.org [11, 12].

### Virtual screening

Structure-based virtual screening in ZINC drug-like database was performed *in silicon*. The structure database was prepared by Schrödinger's LigPrep 2.3 and virtually screened using Schrödinger's Glide 5.5, which incorporated Maestro 9.2 of RHEL 5.0. The docking pocket of PTP-MEG2 for virtual screening was identified by the Receptor Grid-generation tool of Schrödinger, a grid within 5Å from original ligand (B26) in its co-crystal structure (PDB ID: 4GE6).

The increased selectivity of novel inhibitors was introduced in several continuous stages of scaffold optimization by a core-hopping [13, 14] program embedded in Schrödinger Suite 2012. ZINC fragments database [15] was used. All investigated compound candidates were docked into the binding pocket through the flexible docking model. According to the docking scores [16, 17], top candidates with rational structures were classified as potential PTP-MEG2 inhibitors for further study.

### ADME prediction

The “QikProp”[18] module predicted ADME (absorption, distribution, metabolism, and excretion) properties. The parameters of the partition coefficient (QP logPo/w), van der Waals surface area of polar nitrogen and oxygen atoms (PSA), predicted aqueous solubility (QP logS), and apparent MDCK permeability (QPP MDCK) were used in the QikProp to evaluate the acceptability of the compounds [19].

### Chemistry

All starting materials were obtained from commercial suppliers and used without further purification. Building blocks and final products were purified by column chromatography with 200-300 mesh silica gel. ^1^H-NMR spectra was measured by Bruker Avance 400 MHz NMR Spectrometer with TMS as the internal standard and CDCl_3_ as solvent.

#### General procedure for the synthesis of 3a and 3b

The saturated potassium carbonate aqueous solution (55 mmol K_2_CO_3_) was added to a well stirred solution of naphthalen-1-amine (50 mmol) dissolved in 20 mL acetone and cooled to 0°C. Chloroacetyl chloride (55 mmol) was added dropwise. The mixture was slowly warmed to room temperature and stirred until the TLC analysis showed reaction endpoint. The mixture was extracted by ethyl acetate. The organic layer was combined, washed by saturated brine, dried over anhydrous Na_2_SO_4_, filtered, and concentrated to give compound 3a (white solid, 9.0 g, 81.9%). The same method was used to synthesize 3b (white solid, 7.9 g, 63.8%).

#### General procedure for the synthesis of 4a and 4b

A mixture of benzo[d]thiazole-2-thiol (5 mmol), 3a (or 3b) (5 mmol), K_2_CO_3_ (5.5 mmol), and KI (0.5 mmol) in 10 mL DMF was mixed and stirred at room temperature for 4 h. At reaction endpoint, the mixture was extracted by ethyl acetate. The acetate extract was washed with saturated brine, dried over anhydrous Na_2_SO_4_, filtered, and concentrated. The residue was purified by silica gel column chromatography with petroleum ether: ethyl acetate = 7:1-4:1 to obtain the target compound 4a (or 4b).

### Enzymatic assays

Inhibitory activities of compounds 4a and 4b on PTP-MEG2, Protein tyrosine phosphatase 1B (PTP1B) and T cell protein tyrosine phosphatase (TCPTP) were performed with pNPP (Para-Nitro Phenyl Phosphate) as the substrate. Briefly, pNPP was hydrolyzed by PTPs to give para-nitrophenol. Para-nitrophenol converts into para-nitrophenolate (pNP), measured with a spectrophotometer after stopping solution was added. The regents and chemicals used in this assay are as follows: Ni-NTA labeled human recombinant PTPs expressed in E coli; de-phosphorylation assay buffer (50 mM citrate (pH 6.0), 0.1 M NaCl, 1 mM EDTA and 1 mM dithiothreitol (DTT)); substrate (2 mM pNPP) in assay buffer; stopping solution (0.2 M sodium hydroxide). PTPs and test-compounds with geometrically increasing concentrations were mixed in 50 μL buffer in each well of a 96-well plate. An equivalent volume of buffer without PTPs was used as blank control. After pre-incubation for 15 min at room temperature, the substrate (50 μL) was added into each well and incubated at 37°C for 30 min. Stopping buffer was poured into each well on ice and the OD value was read at 405 nm against blank.

### Drug-protein interaction study

The interaction between compound 4a and PTP-MEG2 was predicted by Glide 5.0 of Schrodinger 2012, a commonly used method for docking studies. Crystal structure of PTP-MEG2 (PDB ID: 4GE6) was downloaded from the PDB bank. As a potential inhibitor against PTP-MEG2, compound 4a was prepared *in silicon* via the ligand structure preparation module “LigPrep”. Docking site was determined by original ligand of PTP-MEG2 in its crystal structure.

### Molecular dynamics simulation

Molecular dynamics simulation is a tool used to study the interaction between small molecules and proteins. In this study, we combined the static structures and dynamic information to investigate the binding mode and affinity between PTP-MEG2 and its inhibitors. We used GROMACS 4.5 [20] for Linux for molecular dynamics simulation.

The topology file, partial charges and force field parameters for ligand atoms were generated by the Dundee PRODRG 2.5 Server (University of Dundee, Dundee, Scotland) (beta) [21]. Taking PTP-MEG2 as an example, the simulation system was solvated in a specific box with SPC water solute [22, 23] and sodium and chloride ions were added into the system to neutralize redundant charges. Steepest descents approach was used to minimize energy for the system until reaching a tolerance of 100 kcal/mol. A 40 ns molecular dynamics simulation [24, 25] was performed with a time step of 1 fs, and the corresponding coordinates were stored every 100 fs. All simulations were performed under constant temperature (310 K), periodic boundary conditions and NVT ensembles.

## SUPPLEMENTARY MATERIAL FIGURES AND TABLES


