# Supplementary material for: Virtual screening, optimization, and identification of a novel specific PTP-MEG2 Inhibitor with potential therapy for T2DM
Source: Oncotarget. 2016 Jun 30;7(32):50828–34. doi: 10.18632/oncotarget.10341 (PMC5239439; doi:10.18632/oncotarget.10341)
Supplement: Supplementary file 1 [file oncotarget-07-50828-s001.pdf]

Virtual screening, optimization, and identification of a novel specific PTP-MEG2 Inhibitor with potential therapy for T2DM

Supplementary Material

Table S1

The multiple sequence alignment result of a panel of human protein-tyrosine phosphatases.

PTP	PDB ID	Identity (%)
PTP-MEG2	4GE6	100.0
TCPTP	1L8K	36.9
PTP1B	2QBQ	36.1
PTP-MEG1	2I75	26.6
SHP2	3O5X	25.8
SHP1	3PS5	20.5
LAR	1LAR	18.4
PTPε	2JJD	18.4
LYP	2QCJ	17.0
VHR	1VHR	15.9
PTPμ	2V5Y	13.1
CDC25	1QB0	9.8
PTPγ	2H4V	8.5
DEP1	2DLE	6.7

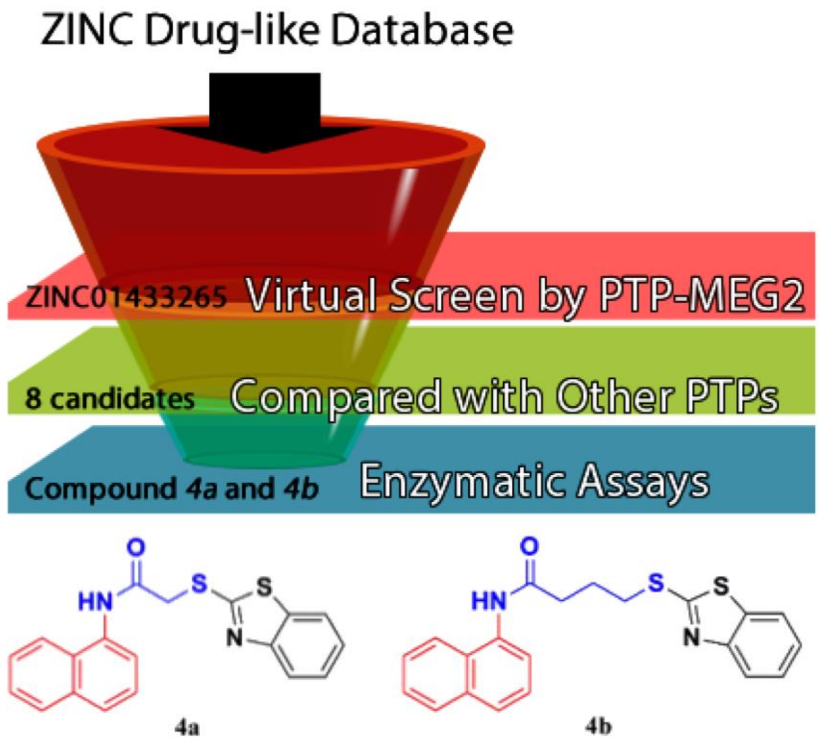

Supplementary Figure1:
